# Comparison of leg length discrepancy after total hip arthroplasty: Direct anterior and posterior lateral approach

**DOI:** 10.1371/journal.pone.0318953

**Published:** 2025-02-25

**Authors:** Yang Lu, Xiang Li, Yihe Hu, Fengchao Zhao

**Affiliations:** Department of Orthopedics, First Affiliated Hospital, Zhejiang University School of Medicine, Hangzhou, China; Tehran University of Medical Sciences Endocrinology and Metabolism Research Institute, IRAN, ISLAMIC REPUBLIC OF

## Abstract

**Background:**

Leg length discrepancy (LLD) after total hip arthroplasty (THA) is a clinical entity that deteriorates clinical outcomes and patients’ satisfaction. Few articles have compared LLD after THA by different surgical approaches.

**Methods:**

A total of 358 consecutive patients who underwent primary THA between January 2016 and November 2018 were retrospectively reviewed. All 4 surgeons performed THA through both direct anterior approach (DAA) and posterior lateral approach (PLA). The primary outcome measurement was LLD. LLD was measured on post-operative anteroposterior bilateral hip radiograph. The secondary outcomes were acetabular abduction, acetabular anteversion, perceived LLD (pLLD) and HHS at 6 weeks, 1 year and 5 years. Intergroup analyses were performed using the *Chi*-square test for enumeration data and the independent sample *t*-test for quantitative data.

**Results:**

There was no inter-group difference in terms of patients’ demographics. The DAA group had decreased LLD compared to the PLA group (3.0 ± 5.9mm vs. 4.2 ± 4.5mm, *p* = 0.027). Meanwhile, the DAA group had a smaller acetabular anteversion than the PLA group (12.9 ± 2.9 vs. 18.4 ± 2.9, *p* < 0.01). At 6-week follow-up, the DAA group had higher HHS (82.2 + 6.2 vs. 80.5 + 6.6, p = 0.015) and less pLLD (P = 0.001) compared to the PLA group.

**Conclusions:**

DAA results in more accurate leg length equalization, reduced pLLD, and improved short-term outcomes compared with PLA.

## Introduction

Despite of the advances, leg length discrepancy (LLD) remains a hurdle which may cause dysfunction, prosthesis failure and bad quality of life. The prevalence of LLD ranges from 4 to 50% in the general population depending on the criterion for clinical significance [1,2]. Post-operative LLD is very common in patients who underwent total hip arthroplasty (THA) and related to a plethora of clinical consequences, like low back pain, dislocation, sciatica and gait disorders [3,4]. Although patients who have functional LLD after THA may achieve partial or complete remission through conservative treatment, general dissatisfaction may still persist [1,4]. With increased importance attached to LLD, clinicians need to take this clinical entity into account throughout the whole perioperative period to minimize its effect. Preoperative templating, intraoperative fluoroscopy, computer navigation and even robotic surgery are increasingly used to enable an equivalent reconstruction of leg lengths [5,6]. However, due to multifactorial socioeconomic issues, these tools are used only in a limited number of medical centers. Most surgeons control LLD based on their own experience without a gold standard.

Different surgical approaches for THA have been debated while limited published data specifically compare the LLD after THA using different approaches. This study evaluated leg lengths following THA performed with direct anterior approach (DAA) and posterior lateral approach (PLA) to determine which approach is more effective in preserving or restoring limb length equality.

## Materials and methods

**Fig 1:**
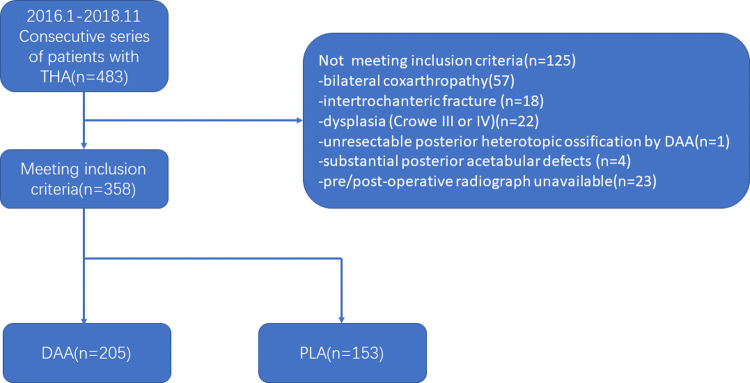
Flow chart diagram of the patient selection process.

The authors retrospectively reviewed all the patients who underwent primary THA between January 2016 and November 2018. All the surgeries were performed by 4 high-volume surgeons from 1 institution. This study is approved by Clinical Research Ethics Committee of the First Affiliated Hospital, Zhejiang University School of Medicine (ZDYY2024-0094). According to our ethics committee, the form of consent is not needed for retrospective study. Data were accessed for research purposes at 26/01/2024. This study included 358 consecutive patients (Fig. 1). Inclusion criteria were patients who underwent primary THA for unilateral coxarthriz, femoral neck fracture, avascular necrosis and dysplasia (Crowe I or II) [7]. Exclusion criteria were patients who have bilateral coxarthropathy, intertrochanteric fracture, dysplasia (Crowe III or IV), posterior heterotopic ossification that cannot be excised through DAA, substantial posterior acetabular defects or pre/post-operative radiograph unavailable. All the surgeons had performed both DAA and THA for more than 8 years. Patients chose the surgical approaches after detailed preoperative conversations. Intra-operative soft-tissue tension, joint stability and leg lengths were checked by shuck tests and leg-to-leg comparison.

**Fig 2:**
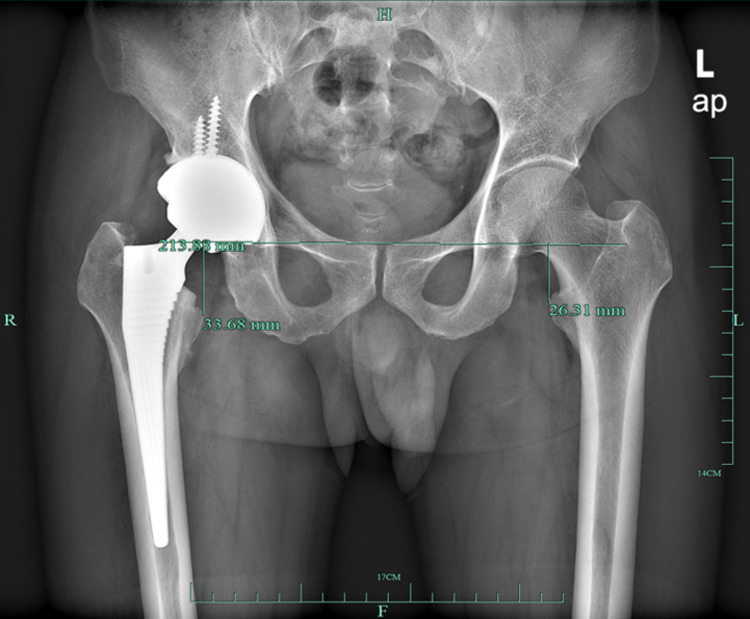
Measurement of leg length discrepancy (LLD) on a standard anteroposterior bilateral hip radiograph. LLD = 33.68mm – 26.31mm = 7.37mm.

**Fig 3:**
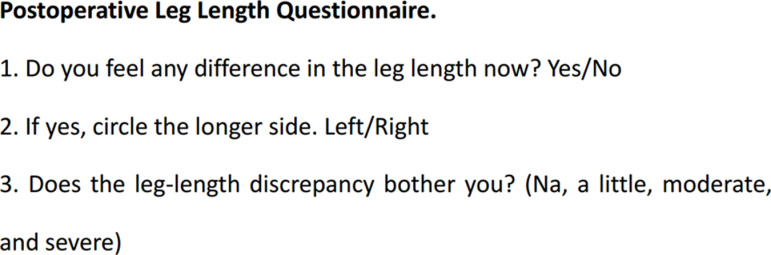
Postoperative Leg Length Questionnaire.

The measurements were made using a Picture Archiving and Communication System (General Electric, IL) monitor by a single observer, who achieved perfect intraobserver and interobserver reliability in our previous study [8]. Limb lengths were measured on a standard anteroposterior bilateral hip radiograph to the nearest 0.01 mm. The LLD was defined as the difference in perpendicular distance between the lower edge of the teardrop to the ipsilateral tip of the lesser trochanter (Fig. 2). Acetabular anteversion was measured using the method described by Pradhan [9]. Harris Hip Score (HHS) questionnaire and a custom-designed questionnaire (Fig. 3) for perceived LLD (pLLD) was administered at 6-week, 1-year and 5-year follow-up.

We had no access to information that could identify any participants during or after data collection. We performed intergroup analyses using the *Chi*-square test for enumeration data, and the independent sample *t* test for quantitative data. Pearson correlation analysis and Logistic regression analysis was used for correlation analysis. A *P-*value <0.05 was considered with statistical significance. We performed all analyses and calculations using SPSS (version 25; IBM, Chicago, IL, USA).

## Results

Table 1 lists the demographic data (S1 Dataset), including age, sex and BMI of the 205 patients who underwent THA through DAA and 153 patients though PLA. There were 158 men and 200 women. The mean age was 59.6 (range, 32 to 85) y mean BMI was 24.8 (range, 18.4 to 29.6). The demographic data were comparable between DAA and PLA. The operative time of DAA were longer than PLA (85.6 ± 26.1min vs. 79.2 ± 18.2min, p = 0.010).

**Table 1:** Patient demographics by different approach.

		DAA	PLA	*P* value
Age(y)		60.4 ± 9.9	58.6 ± 11.9	0.133
Sex	men	85 120	73 80	0.142
	women			
BMI		24.6 ± 2.2	24.9 ± 2.1	0.149

### Radiographic measurement

The mean LLD, acetabular abduction and anteversion were 3.5 mm (range, -14.9 to 18.6 mm), 45.3°(range, 33.8 to 58.9°) and 15.2°(range, 8.2 to 23.7°), respectively. LLD was increased in the PLA group compared to the DAA group (4.2 ± 4.5mm vs. 3.0 ± 5.9mm, *p* = 0.027). When LLD of more than 5mm or 10mm was set as an outlier, the two groups showed no difference in distribution. Acetabular abduction was not different between the two groups. The PLA group had a larger acetabular anteversion than the DAA group (18.4 ± 2.9 vs. 12.9 ± 2.9, *p* < 0.01) (Table 2).

**Table 2:** Radiographic measurement data by different approaches.

	DAA	PLA	*P* value
LLD(mm)	3.0 ± 5.9	4.2 ± 4.5	0.027
≥5 <5 ≥10 <10	81	68	0.204
	124	85	
	18	10	0.282
	187	143	
Acetabular abduction(°)	45.1 ± 4.0	46.5 ± 4.3	0.431
Acetabular anteversion(°)	12.9 ± 2.9	18.4 ± 2.9	<0.01

### PLLD

At 6 weeks, 83/358 (23.2%) patients reported LLD, and 16 patients complained of a little discomfort and 4 patients complained of moderate discomfort. At 6 weeks, patients who underwent THA through PLA were more likely to perceive LLD compared to DAA (P = 0.001) (Table 3).

**Table 3:** pLLD by different approaches.

	pLLD	DAA	PLA	*P* value
6 weeks	Y	35	48	0.001
	N	170	105	
1 year	Y	13	19	0.052
	N	179	133	
5 years	Y	10	13	0.140
	N	170	126	

At 1 year (14 loss), 32/344 (9.3%) patients perceived LLD, and 3 of the 32 patients did not report LLD at 6 weeks. At 1 year, 9 patients complained of a little discomfort and 3 patients complained of moderate discomfort.

At 5 years (39 loss), 23/319 (7.2%) patients perceived LLD, and 4 patients complained of a little discomfort. No patient complained of moderate discomfort. Two of the 3 patients who did not perceive LLD at 6 weeks but perceived LLD at 1 year still perceived LLD at 5 years.

There was no difference of pLLD between DAA and PLA at 1 year and 5 years. pLLD was not correlated with radiographic LLD at 6-week (P = 0.118), 1-year (P = 0.427) and 5-year follow-up (*P* = 0.969).

### Clinical outcome

At 6-week follow up, the DAA group achieved higher HHS compared to the PLA group (82.2 ± 6.2 vs. 80.5 ± 6.6, *p* = 0.015) (Table 4). HHS was comparable between DAA and PLA at 1-year and 5-year follow up. When LLD≤0, LLD was not correlated with HHS at 6 weeks (*P* = 0.171), 1 year (*P* = 0.780) and 5 years (*P* = 0.781). When LLD>0, a weak negative correlation was observed between LLD and HHS at 6 weeks (*P* = 0.010) but not observed at 1 year (*p* = 0.505) and 5 years (*P* = 0.323). HHS was not different between patients who perceived and did not perceive LLD at 6 weeks (P = 0.662), 1 year (P = 0.057) and 5 years (P = 0.124).

**Table 4:** HHS by different approaches.

	DAA	PLA	*P* value
6-week	82.2 ± 6.2	80.5 ± 6.6	0.015
1-year	90.2 ± 5.1	89.8 ± 5.5	0.561
5-year	90.1 ± 5.0	90.2 ± 5.4	0.897

## Discussion

Inequality of lower extremities leads to altered gait kinematics, lower active range of motion and worse clinical outcome [4,10]. LLD can also induce polyethylene wear which may cause aseptic loosening and finally lead to revision surgery [11,12]. Although LLD ≤ 20 mm may not have any detected effect on muscle strength, extra rehabilitation effort and conservative treatment is required [13].

Postoperative leg length equality is also a major concern for the patient. PLLD is one of the most frequent reasons for litigation [14] which is more complicated and multifactorial than objective (measured/true) LLD. In our study, pLLD did not correlate with radiographic LLD. PLLD is more likely to be patient specific and both psychological and physiological factors might play a significant role. PLLD becomes more frequent in patients who underwent both THA and a spinal fusion [15]. On the other hand, pre-existing pelvic declination and lumbar scoliosis may cause pLLD [1]. Some authors argued that pLLD will alleviate over time [1], while others suggested the opposite [4]. In our study, half of the patients who perceived LLD at 6 weeks remitted at 1-year follow-up. This is consistent with findings of Adams et al. [16] our study had a larger cohort and a longer follow-up time of at least five years. Between 1 year and 5 years patients’ perception of pLLD remained almost unchanged but the subjective negative effect of pLLD reduced persistently. It was interesting that Adams et al introduced postoperative change in limb length (△*L*). They found that patient’s perception of LLD correlates with the amount of surgical lengthening. They also demonstrate worse prognosis with increased LLD because pLLD improved more over time in patients who had decreased LLD. Our study included patients who had femoral fracture, so we could not study △*L.* We found that pLLD was not correlated with radiographic LLD, which suggest that pre-existing LLD played an important role in pLLD. Konyves et al Pointed out that patients with longer leg were more likely to detect LLD than those who had short or equal leg lengths [4]. We did not find pLLD relate to shortening or lengthening of the leg. However, we found that HHS correlated with the magnitude of leg lengthening at 6 weeks, indicating that LLD negatively affect prognosis. These findings may help clinicians better understand the natural history and clinical influences of pLLD.

Restoration of normal hip biomechanics is a key goal for hip surgeons and maintaining the leg length remains a challenge. The factors that contribute to LLD can be divided into two categories, immutable and mutable factors. The immutable factors are patients’ physical structures like morphology of femoral medullary cavity [17], soft-tissue tension, pre-existing LLD and offset heterogeneity. These factors cannot be altered during the operation. The mutable factors are types of prostheses, the position of the cup, osteotomy level and size/length of femoral component. Once the cup is fixed and type of the prosthesis is already chosen, it is difficult to maintain both leg lengths and offset, and at the same time ensure stability. Therefore, it is essential to prudently read the pre-operative pelvic radiograph, evaluate and quantify all mutable and immutable factors, better with templating. The relative importance of the malposition of acetabular and femoral components as causes of LLD is debated [4,17,18]. Tripuraneni et al pointed out that based on execution of preoperative templating, acetabular malposition contributed the most to LLD [18]. Inversely, Konyves et al believed that the femoral part of the operated joint affected LLD more [4]. We think this paradox can be attributed to whether preoperative templating was used. The literature mentioned different ways of intra-operative comparison of leg lengths, like usage of pins and compass-like devices, measurement of the trochanteric/joint ratio and ﬂuoroscopy, etc. The most economical and frequently used methods are still the shuck tests and leg-to leg comparison of the lower extremities based on bony marks.

Our institution has been using both DAA and PLA for THA since 2010. PLA is the classical and most frequently used approach for THA with good femoral exposure and preservation of the abductors [19]. DAA has gained increased popularity since the second decade of 20th century which is accomplished without detaching any of the muscles or tendons from the pelvis or femur [20–22]. The dislocation rate and periprosthetic joint infection rate of the two approaches are not different [20,23,24]. However, DAA was found to be associated with a higher incidence of lateral femoral cutaneous nerve injury and superficial infection [24,25]. In our study, HHS was higher in the DAA group at 6 weeks, showing better short-term outcome. This is consistent with the recent literature that DAA is related to a quicker functional recovery, shorter hospital stay and an earlier return to daily activities compared with PLA [25,26]. DAA is associated with a shorter and inconspicuous incision, but longer operative time and steeper learning curve [26]. in our study, the prolonged operative time of DAA was mainly related to the time spent on femur exposure.

Debi et al surveyed a cohort of 172 patients who underwent THA by a single surgeon. They found that the DAA improved leg length equality compared to the anterior lateral approach [27]. Nam et al compared 270 patients and found no difference of LLD among the DAA, PLA, and navigated PLA. In the study of Nam et al, THA by three different approaches are performed by three joint surgeons respectively which will introduce a ineluctable bias [28]. In our study, we compared leg length after primary THA conducted by four senior doctors between DAA and PLA. We found that DAA was better for restoring lengths of the lower limbs. Among the factors that affect surgeons’ decision making, firm implant fixation and joint stability take precedence over equal leg lengths. We think one reasonable explanation of our finding is that surgeons tend to lengthen the femoral neck and enlarge acetabular anteversion to improve posterior stability when using PLA. However, DAA protect the posterior structure, which cut down the worry for a posterior dislocation. The relatively decreased LLD by anterior approach compared with traditional approaches may also be attributed to that the supine position allows for intraoperative ﬂuoroscopy and more accurate leg-to-leg comparison of leg lengths [22,29]. Researches showed that leg-to-leg comparison of leg length in the lateral position is misleading due to the adduction and rotation of the operated extremity [30]. As far as we know, this is the first study to compare pLLD after THA between different approaches. We found that patients who underwent THA through DAA were less likely to perceive LLD. We attribute this to the quick recovery and minimal-invasive nature of DAA.

The weakness of this study includes its retrospective nature. the demographics were comparable between the two groups but the surgical approaches were not randomized which will introduce selection bias. Meanwhile, the measurements were performed on anteroposterior bilateral hip radiographs, which are subject to postural and magnification error. The position of the femur can also affect the accuracy of radiographic measurements. Although all the patients recruited are from high-volume senior doctors, it is still hard to eliminate heterogeneous factors of surgeons’ preference of types of implant and other clinical experiences.

## Conclusion

DAA results in more accurate leg length equalization, reduced pLLD, and improved short-term outcomes compared with PLA.

## Supporting information

S1 Dataset Database (XLSX)
